# From Pancytopenia to Hyperleukocytosis, an Unexpected Presentation of Immune Reconstitution Inflammatory Syndrome in an Infant with Methylmalonic Acidemia

**DOI:** 10.3390/children11080990

**Published:** 2024-08-14

**Authors:** Samuel Sassine, Amandine Remy, Tanguy Demaret, François Proulx, Julie Autmizguine, Fatima Kakkar, Thai Hoa Tran, Caroline Laverdière, Ellery T. Cunan, Catalina Maftei, Grant Mitchell, Hélène Decaluwe, Jade Hindié

**Affiliations:** 1 CHU Sainte-Justine Research Center, Montreal, QC H3T 1C5, Canada; samuel.sassine@umontreal.ca (S.S.); francois.proulx1@umontreal.ca (F.P.); julie.autmizguine@umontreal.ca (J.A.); fatima.kakkar@umontreal.ca (F.K.); thai.hoa.tran@umontreal.ca (T.H.T.); caroline.laverdiere@umontreal.ca (C.L.); grant.mitchell@umontreal.ca (G.M.); helene.decaluwe@umontreal.ca (H.D.); 2 Faculty of Medicine, Université de Montréal, Montreal, QC H3T 1J4, Canada; amandine.remy.med@ssss.gouv.qc.ca (A.R.); tanguy.demaret@umontreal.ca (T.D.); catalina.maftei@umontreal.ca (C.M.); 3 Division of General Pediatrics, Department of Pediatrics, CHU Sainte-Justine, Montreal, QC H3T 1C5, Canada; 4 Division of Genetics, Department of Pediatrics, CHU Sainte-Justine, Montreal, QC H3T 1C5, Canada; 5 Division of Pediatric Critical Care, Department of Pediatrics, CHU Sainte-Justine, Montreal, QC H3T 1C5, Canada; 6 Division of Infectious Diseases, Department of Pediatrics, CHU Sainte-Justine, Montreal, QC H3T 1C5, Canada; 7 Division of Hematology-Oncology, Department of Pediatrics, Charles-Bruneau Cancer Center, CHU Sainte-Justine, Montreal, QC H3T 1C5, Canada; 8 Division of Pediatric Critical Care, Department of Pediatrics, Montreal Children’s Hospital, McGill University, Montreal, QC H3A 0G4, Canada; ellery.cunan@mail.mcgill.ca; 9 Division of Immunology and Rheumatology, Department of Pediatrics, CHU Sainte-Justine, Montreal, QC H3T 1C5, Canada

**Keywords:** methylmalonic acidemia, pancytopenia, hyperleukocytosis, immune reconstitution inflammatory syndrome, cytomegalovirus, *Pneumocystis jirovecii*

## Abstract

A 2.5-month-old girl admitted for failure to thrive and severe pancytopenia was diagnosed with methylmalonic acidemia (MMA) secondary to transcobalamin II deficiency, an inborn error of vitamin B12 metabolism. Opportunistic Cytomegalovirus and *Pneumocystis jirovecii* pneumonia led to severe acute respiratory distress syndrome (ARDS) and immune reconstitution inflammatory syndrome (IRIS) after treatment initiation with vitamin B12 supplementation. In children with interstitial pneumonia-related ARDS, normal lymphocyte count should not delay invasive procedures required to document opportunistic infections. MMA can be associated with underlying lymphocyte dysfunction and vitamin B12 supplementation can fully reverse the associated immunodeficiency. IRIS may appear in highly treatment-responsive forms of pancytopenia in children and prompt treatment of dysregulated inflammation with high-dose corticosteroids should be initiated.

## 1. Introduction

Pancytopenia is of rare occurrence in pediatric populations [1] and inborn errors of metabolism (IEMs) may be overlooked in the broad and extensive differential diagnosis [2,3,4,5]. Pancytopenia may be the first manifestation of several IEMs including methylmalonic acidemias (MMAs) [6]. It is caused by methylmalonylCoA mutase deficiency or activation disorder of its cofactor (vitamin B12) [7]. Vitamin B12 undergoes a series of activation reactions to form methylcobalamin and adenosylcobalamin. These reactions involve various enzymes, which can suffer mutations resulting in the dysfunction of either or both methylcobalamin and adenosylcobalamin. The implications of these mutations are serious, as methylcobalamin and adenosylcobalamin are, respectively, coenzymes of Methionine Synthase and Methylmalonic-CoA Mutase. The role of Methionine Synthase is to convert homocysteine into methionine. Methylmalonic-CoA Mutase transforms l-Methylmalonyl-CoA into Succinyl-CoA, which is a substrate of the Krebs cycle. l-Methylmalonyl-CoA generates methylmalonic acid as it accumulates, causing MMA [7]. Vitamin B12 activation is then mediated by several enzymes and proteins, of which transcobalamin II (TCN2), a membrane transport protein, is important for vitamin B12 intracellular uptake [8].

Patients with TCN2 deficiency, regardless of mutation types, classically present before 1 year of age with failure to thrive, digestive symptoms and recurrent infections secondary to pancytopenia [9,10,11,12]. Innate and adaptative immunity dysfunction has been reported in TCN2 deficiency [11,13,14,15,16]. Those children show an excellent bio-clinical response to vitamin B12 supplementation (under the form of hydroxocobalamin) [11,16,17,18].

We report the case of an infant with TCN2 deficiency who presented severe pancytopenia and long-lasting acute respiratory distress syndrome (ARDS) secondary to immune reconstitution inflammatory syndrome (IRIS) and combined Cytomegalovirus (CMV) and *Pneumocystis jirovecii* (PJ) infection.

## 2. Case Presentation

A 2.5-month-old infant was admitted for failure to thrive and pancytopenia. She was born at term with a birthweight of 2800 g (21st percentile) from consanguineous West African parents. Newborn urine screening (carried out in all children in Quebec, Canada [19]) showed mild methylmalonic aciduria (urine MMA 623 mmol/mol of creatinine, referral threshold: 250). At 2.5 months of age, a medical visit at the IEM reference center led to hospitalization for dehydration, weakness and severe pancytopenia. Physical examination was unremarkable apart from failure to thrive (weight inferior to first percentile) and hypotonia.

**Figure 1:**
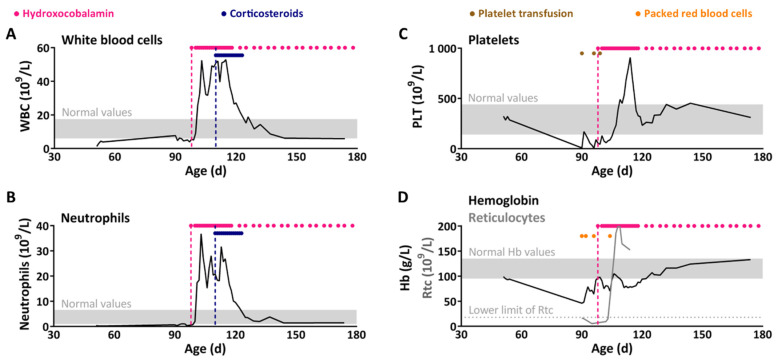
After hydroxocobalamin treatment, the white blood cell (WBC) count dramatically increased and the respiratory status of the patient deteriorated, both compatible with immune reconstitution inflammatory syndrome (IRIS). One month after hydroxocobalamin treatment, coupled with a 2 week corticosteroid course, complete blood count normalized. White blood cells (**A**) and neutrophils (**B**) responded dramatically to hydroxocobalamin treatment. Platelets (**C**) normalized after a delayed overshoot. Hemoglobin (**D**) slowly normalized after a rapid increase in reticulocytes (**D**) (Rtc, gray curve) soon after hydroxocobalamin injection.

Complete blood count showed hemoglobin 46.0 g/L (N (normal values) 95.0–135.0), reticulocytes 16.5 × 10^9^/L (N 18.0–158.0), mean corpuscular volume 86.9 fL (N 77.0–115.0 fL), platelets 8.0 × 10^9^/L (N 140.0–440.0), absolute neutrophil count 0.7 × 10^9^/L (N 1.0–6.6) and absolute lymphocyte count 6.8 × 10^9^/L (N 4.0–10.6) (Figure 1). The initial blood smear did not show blasts. Viral infectious investigations revealed Respiratory Syncytial virus in nasopharyngeal secretions, negative parvovirus B19 PCR in blood and negative CMV PCR in urine. Bacterial cultures were all negative. Baseline albumin, TSH, ferritin, folate, LDH, coagulation profile, haptoglobin, liver and renal function were normal. Metabolic investigations confirmed elevated MMA in plasma (33.6 µM; N < 0.5) and urine (1129 mmol/mol creatinine, N < 10) without metabolic decompensation (pH: 7.38 (N 7.34–7.44), anion gap: 6.4 mmol/L (N 4–12), ammonia 40 µM (N 5–55)). Serum vitamin B12 was normal (657 pM, N 191–1163). Bone marrow aspiration excluded malignancy and revealed near-absence of erythroid precursors, myelodysplasia, megaloblastosis and less than 10% of myeloblasts, suggestive of inherited defects of vitamin B12 metabolism.

**Figure 2:**
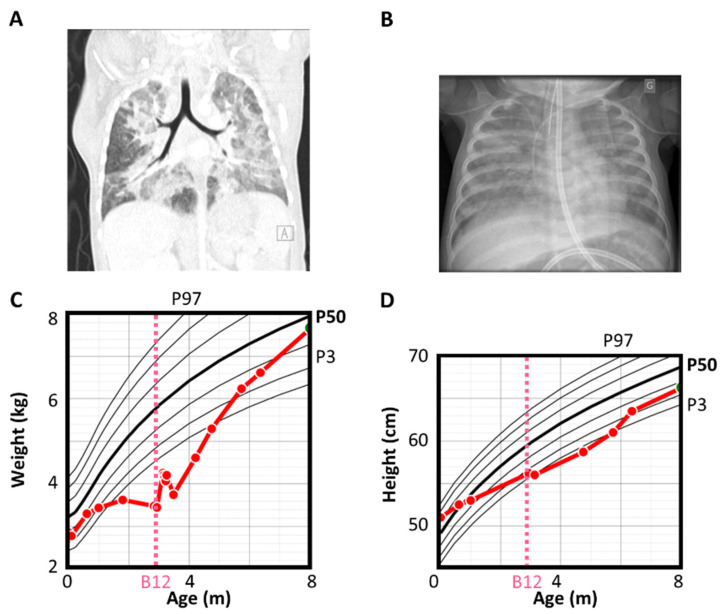
Patients X-ray, CT-scan and growth charts. Chest CT-scan confirming severe interstitial lung disease (**A**), chest X-ray showing interstitial lung disease (**B**), patient weight (**C**) and height (**D**) growth chart. The red lines represent the patient’s curve.

Daily intramuscular vitamin B12 (1 mg) injections were initiated on treatment day 1 (D1). On D3, the child developed acute respiratory failure with marked hypoxia simultaneously with hyperleukocytosis (52 × 10^9^/L): neutrophilia up to 28 × 10^9^/L, lymphocytosis up to 23 × 10^9^/L and monocytosis up to 10.3 × 10^9^/L (Figure 1). Chest X-ray and chest CT-scan confirmed severe interstitial lung disease (Figure 2). On D5, the patient required endotracheal intubation and mechanical ventilation due to ARDS. Baseline immunological investigations were normal, including immunoglobulin levels (IgA: 0.9 g/L, IgM: 7.8 g/L), complement components (C3, C4, CH50) and lymphocytes immunophenotyping (Table S1). HIV test was negative. Results were not consistent with hemophagocytic lymphohistiocytosis. Flow cytometry ruled out leukemia and cytogenetic studies confirmed normal karyotype and absence of clonal chromosomal abnormalities.

Empiric antimicrobial treatment with fluconazole, piperacillin/tazobactam and trimethoprim/sulfamethoxazole was started on D4 after bronchoalveolar lavage was performed. Bronchoalveolar PCR test results revealed cytomegalovirus (CMV, 4074 copies/mL) on D9 and *Pneumocystis jirovecii* (PJ, 14,976,000 copies/mL) on D13. Ganciclovir and anti-CMV immunoglobulins were initiated on D9. On D14, as the child’s respiratory function still required significant ventilatory support despite appropriate anti-viral and anti-PJ therapy (including prednisone), high dose intravenous corticosteroids were initiated (4 mg/kg/day) with the hypothesis of a contributing immune reconstitution inflammatory syndrome (persisting leukocytes up to 50 × 10^9^/L). The child’s respiratory function finally improved, and she was weaned off respiratory support on D17. Corticosteroid therapy was slowly weaned and ended on D26. Complete blood count normalized on D39.

Rapid whole genome sequencing revealed a homozygous pathogenic variant in *TCN2* gene (c.580+1G>C, suspected splicing defect, NM_000355.4 [18]). Under vitamin B12 treatment she was not considered immunodeficient anymore and received, without complication, live vaccines at 1 year of age.

## 3. Discussion

We report the first case of long-lasting ARDS secondary to IRIS and combined CMV and PJ pulmonary opportunistic infections after treatment in a patient with TCN2 deficiency, in contrast to previously reported cases of children with TCN2 deficiency who exhibited excellent clinical outcomes following treatment with vitamin B12 [11,13,14,15,16,17,18].

The mechanisms underlying immunodeficiency in MMA are not fully understood, but case series of children with TCN2 deficiency have reported severe opportunistic infections including PJ pneumonia. Decreased IgG levels were identified in numerous cases and some patients had low T- and B-cell counts, inverted CD4/CD8+ T-cell ratio and low natural killer cell counts, all which responded well to therapy [11,13,14,15,16]. All these lymphocyte count and immunoglobulin level abnormalities were not present in our patient. Seger et al. reported improvement in the destructive capacity of polymorphonuclear leukocytes after B12 infusion among patients with TCN2 deficiency [20]. Intracellular vitamin B12 depletion, such as in MMA, generates leukocyte dysfunctions other than DNA replication impairment. Therefore, in children with interstitial pneumonia-related ARDS, especially in those with suspected IEM, normal lymphocyte count and immunophenotyping should not delay more invasive procedures in search of opportunistic infections or empiric treatment [20].

The child’s clinical improvement and the resolution of hyperleukocytosis following high-dose corticosteroids makes the hypothesis of IRIS likely in the context of active infection and a highly treatment-responsive form of pancytopenia and immune cell dysfunction. IRIS was described in patients with human immunodeficiency virus (HIV) initiating antiretroviral therapy (ART) in the context of active infection, especially PJ [21,22]. The rapid release of CD4+ lymphocytes following the start of ART leads to the development of IRIS and corticosteroids have been shown to be effective in treating this condition in adults and children [21,22]. Pathophysiological mechanisms of IRIS include the recovery of pathogen-specific immunity coupled with a high antigenic burden accumulated during the period of immunosuppression leading to exaggerated immune activation [22,23]. In our patient, the large antigenic burden induced by RSV, CMV and PJ thus triggered IRIS after vitamin B12 treatment which reversed immunosuppression [22,23,24].

IRIS is also a known phenomenon in oncology in children recovering from chemotherapy and after stem cell transplant [25,26] To our knowledge, IRIS in children with IEM such as TCN2 deficiency has not been reported. Other cases of IRIS induced by highly treatment-responsive forms of pancytopenia have been described. Shima et al. and Arakawa et al. reported that treatment of methotrexate-induced pancytopenia with folate, associated with concomitant PJ and CMV infection, can lead to severe IRIS [27,28]. Vitamin B12 and folate deficiencies prevent DNA replication via the same tetrahydrofolate activation pathway [29]. TCN2 deficiency prevents vitamin B12 from entering the cell and methotrexate blocks the intracellular reaction chain which depends on vitamin B12 and folate. IRIS induced by the reversal of these conditions in the context of active infection can therefore be explained by a similar pathophysiological process.

In conclusion, IEM, such as TCN2 deficiency, should be considered in infantile pancytopenia. In children with interstitial pneumonia-related ARDS, normal lymphocyte count should not delay invasive procedures required to document opportunistic infections. MMA can be associated with underlying lymphocyte dysfunction and vitamin B12 supplementation can fully reverse the associated immunodeficiency. IRIS may appear in highly treatment-responsive forms of pancytopenia in children and prompt treatment of dysregulated inflammation with high-dose corticosteroids should be rapidly initiated.

## Data Availability

The original contributions presented in the study are included in the article/Supplementary Material, further inquiries can be directed to the corresponding author.

## Supplementary Materials

The following supporting information can be downloaded at: https://www.mdpi.com/article/10.3390/children11080990/s1, Table S1: Lymphocytes immunophenotyping values. Reference [30] is cited in the supplementary materials.

## References

[1] Pine M., Walter A.W. (2010). Pancytopenia in hospitalized children: A five-year review. J. Pediatr. Hematol. Oncol..

[2] Gnanaraj J., Parnes A., Francis C.W., Go R.S., Takemoto C.M., Hashmi S.K. (2018). Approach to pancytopenia: Diagnostic algorithm for clinical hematologists. Blood Rev..

[3] Weinzierl E.P., Arber D.A. (2013). The differential diagnosis and bone marrow evaluation of new-onset pancytopenia. Am. J. Clin. Pathol..

[4] Devitt K.A., Lunde J.H., Lewis M.R. (2014). New onset pancytopenia in adults: A review of underlying pathologies and their associated clinical and laboratory findings. Leuk. Lymphoma.

[5] Mudenge B., Savage D.G., Allen R.H., Gangaidzo I.T., Levy L.M., Gwanzura C., Moyo A., Kiire C., Mukiibi J., Stabler S.P. (1999). Pancytopenia in Zimbabwe. Am. J. Med. Sci..

[6] De Lonlay P., Fenneteau O., Touati G., Mignot C., de Villemeur T.B., Rabier D., Blanche S., de Baulny H.O., Saudubray J.M. (2002). Manifestations hématologiques dans les erreurs innées du métabolismeHaematological manifestations of inborn errors of metabolism. Arch. Pediatr..

[7] David Watkins D.S.R. (2022). Inherited defects of cobalamin metabolism. Vitam. Horm..

[8] Quadros E.V., Sequeira J.M. (2013). Cellular uptake of cobalamin: Transcobalamin and the TCblR/CD320 receptor. Biochimie.

[9] Trakadis Y.J., Alfares A., Bodamer O.A., Buyukavci M., Christodoulou J., Connor P., Glamuzina E., Gonzalez-Fernandez F., Bibi H., Echenne B. (2014). Update on transcobalamin deficiency: Clinical presentation, treatment and outcome. J. Inherit. Metab. Dis..

[10] Nashabat M., Maegawa G., Nissen P.H., Nexo E., Al-Shamrani H., Al-Owain M., Alfadhel M. (2017). Long-term Outcome of 4 Patients With Transcobalamin Deficiency Caused by 2 Novel TCN2 Mutations. J. Pediatr. Hematol. Oncol..

[11] Kose E., Besci O., Gudeloglu E., Suncak S., Oymak Y., Ozen S., Isguder R. (2020). Transcobalamin II deficiency in twins with a novel variant in the TCN2 gene: Case report and review of literature. J. Pediatr. Endocrinol. Metab..

[12] Hakami N., Neiman P.E., Canellos G.P., Lazerson J. (1971). Neonatal megaloblastic anemia due to inherited transcobalamin II deficiency in two siblings. N. Engl. J. Med..

[13] Wong S.N., Low L.C., Lau Y.L., Nicholls J., Chan M.Y. (1992). Immunodeficiency in methylmalonic acidaemia. J. Paediatr. Child. Health.

[14] Watkins D., Rosenblatt D.S. (2020). Immunodeficiency and inborn disorders of vitamin B12 and folate metabolism. Curr. Opin. Clin. Nutr. Metab. Care.

[15] Yang C., Wang X., Wang C., Zhang X., Li Y., Yu Y., Liu S. (2021). Clinical and genetic analysis of a child with transcobalamin II deficiency. Zhonghua Yi Xue Yi Chuan Xue Za Zhi.

[16] Zhan S., Cheng F., He H., Hu S., Feng X. (2020). Identification of transcobalamin deficiency with two novel mutations in the TCN2 gene in a Chinese girl with abnormal immunity: A case report. BMC Pediatr..

[17] Martino F., Magenta A., Troccoli M.L., Martino E., Torromeo C., Putotto C., Barilla F. (2021). Long-term outcome of a patient with Transcobalamin deficiency caused by the homozygous c.1115_1116delCA mutation in TCN2 gene: A case report. Ital. J. Pediatr..

[18] Schiff M., Ogier de Baulny H., Bard G., Barlogis V., Hamel C., Moat S.J., Odent S., Shortland G., Touati G., Giraudier S. (2010). Should transcobalamin deficiency be treated aggressively?. J. Inherit. Metab. Dis..

[19] Québec G.D. Blood and Urine Screening in Newborns. https://www.quebec.ca/en/health/advice-and-prevention/screening-and-carrier-testing-offer/blood-and-urine-screening-newborns.

[20] Hitzig W.H., Fràter-Schröder M., Seger R. (1979). Immunodeficiency due to transcobalamin I1 deficiency. Enzyme Defects and Immune Dysfunction.

[21] Shelburne S.A., Hamill R.J., Rodriguez-Barradas M.C., Greenberg S.B., Atmar R.L., Musher D.W., Gathe J.C., Visnegarwala F., Trautner B.W. (2002). Immune reconstitution inflammatory syndrome: Emergence of a unique syndrome during highly active antiretroviral therapy. Medicine.

[22] Boulware D.R., Callens S., Pahwa S. (2008). Pediatric HIV immune reconstitution inflammatory syndrome. Curr. Opin. HIV AIDS.

[23] Sun H.Y., Singh N. (2009). Immune reconstitution inflammatory syndrome in non-HIV immunocompromised patients. Curr. Opin. Infect. Dis..

[24] Openshaw P.J., Tregoning J.S. (2005). Immune responses and disease enhancement during respiratory syncytial virus infection. Clin. Microbiol. Rev..

[25] Lei J.Y., Chen H., Zhou D.H., Xu L.H., Fang J.P., Mai Y.G. (2022). *Pneumocystis jirovecii*-associated immune reconstitution inflammatory syndrome-like phenomenon in a child with leukaemia: A case report and literature review. BMC Pediatr..

[26] Liu S., Huo F., Dai G., Wu J., Qin M., Mao H., Wang Q. (2022). Case Report: Immune reconstitution inflammatory syndrome after hematopoietic stem cell transplantation for severe combined immunodeficiency. Front. Immunol..

[27] Shima N., Kokuzawa A., Saito K., Kamata Y., Nagashima T., Sato K. (2022). Immune Reconstitution Inflammatory Syndrome Associated with *Pneumocystis jirovecii* Pneumonia and Cytomegalovirus Colitis in a Patient with Rheumatoid Arthritis. Intern. Med..

[28] Arakawa N., Eguchi K., Nakamura Y., Tsukahara Y., Koushima Y., Matsushima H. (2021). Immune Reconstitution Inflammatory Syndrome-like Condition Associated with *Pneumocystis jirovecii* Pneumonia During Folinic Acid Treatment in a Rheumatoid Arthritis Patient. Intern. Med..

[29] Hariz A., Bhattacharya P.T. (2023). Megaloblastic Anemia. StatPearls [Internet].

[30] Comans-Bitter W.M., de Groot R., van den Beemd R., Neijens H.J., Hop W.C., Groeneveld K., Hooijkaas H., van Dongen J.J.M. (1996). Immunophenotyping of blood lymphocytes in childhood. J. Pediatr..

