# Supplementary material for: From Pancytopenia to Hyperleukocytosis, an Unexpected Presentation of Immune Reconstitution Inflammatory Syndrome in an Infant with Methylmalonic Acidemia
Source: Children (Basel). 2024 Aug 14;11(8):990. doi: 10.3390/children11080990 (PMC11352300; doi:10.3390/children11080990)
Supplement: Supplementary file 1 [file children-11-00990-s001.zip › children-3125278-supplementary.pdf]

Table S1. Lymphocytes immunophenotyping values.

	Patients Value	Normal Value [30]
Complete lymphocyte count (10^6/L)	13,000	3700–9600
CD3+ T-cells count (10^6/L)	11,050	2300–6500
CD4+ T-cells count (10^6/L)	8450	1500–5000
CD8+ T-cells count (10^6/L)	1820	500–1600
B-cells count (CD19+ cells count) (10^6/L)	1404	600–3000
